# Acknowledgment to the Reviewers of *Clinics and Practice* in 2022

**DOI:** 10.3390/clinpract13010008

**Published:** 2023-01-12

**Authors:** Clinics and Practice Editorial Office

**Affiliations:** MDPI AG, St. Alban-Anlage 66, 4052 Basel, Switzerland

High-quality academic publishing is built on rigorous peer review. *Clinics and Practice* was able to uphold its high standards for published papers due to the outstanding efforts of our reviewers. Thanks to the efforts of our reviewers in 2022, the median time to first decision was 23 days and the median time to publication was 49 days. Regardless of whether the articles they examined were ultimately published, the editors would like to express their appreciation and thank the following reviewers for the time and dedication that they have shown *Clinics and Practice*:
Abbas MardaniLuciana MontenegroAbdallah El-Sayed AllamLudmila KazdovaAbdolkarim HosseiniLuigi BennardoAbdulqadir J. NashwanLuis Enrique Juárez-VillegasAboElenain MansourŁukasz ZadrożnyAgustín OterinoMaeirah AshaieAhmed Abdelbaset-IsmailMagdalena KostrzewaAkihito YamauchiMalin L. NordingAl OzonoffManish DhawanAleksander LukasiewiczManoel J TeixeiraAleš BlincManoranjan D'SouzaAlessandro MianiMarco CascellaAlessandro TavelliMaria Delia Perez-MontielAlexander HaraganMaria MazzitelliAlexandru VlasaMaria Rita BiancoAli AkilMariia NesterkinaAlice Chiara ManettiMarija M. BožićAlice VerticchioMarilena IanculescuAlper SozutekMarko BaškovićAmaniel KefleyesusMarta KopańskaAmelia BrunaniMarta NardiniAna Šešelja-PerišinMarwa GaberAnastasios S. TsarouchasMattia Russel PantaloneAndrew ChanMauricio Rodriguez-dorantesAndy Wei-Ge ChenMaurizio G. AbrignaniAngela IshakMaxwell SuAntonio Jesús Martínez-OrtegaMd Rabiul IslamAntonio Jesús Molina FernándezMichela CinquiniArtur SłomkaMiguel Castelo-Branco SousaArturo Sanchez-PerezMiguel Silva-RamosAthanasios KiourtisMiruna NemeczAthanassios KyrgidisMirza PojskicAyaz ShahidMititelu-Tartau LilianaAyman ElnahriMohamed ElnaemBarbara GordonMohammad A. SaadBeatriz Álvarez-EmbarbaMohammed GrawishBiagio PincheraMoien AB KhanBianca E. ŐszMónica Ríos-SilvaBrian BuchananMonika KniotekCalvin YeangMoshe RubinCan CuiMuhammad AqeelCarlo CampanaMuhammad Hammad ButtCarmen SolcanMukhtar AnsariCarol BurnsMustafa TascanovCaterina De LucaNan YangCeleste ManfrediNaoki UmemuraChen ChenNatasa PopovicChendong HanNicolae GicaChristian JansenNiels QvistChristopher LoNimrat ChatterjeeClaudia Ramírez-RenteríaNiroj Kumar SahooClaudia TodaroOlga SeifertClaudio De LuciaÖzgül DüzgünConcetta Di NoraPalanivel ChinnakaliCristi TartaPaolo Albino FerrariCristiana Furtado FirminoPathiyil Ravi ShankarDaisy VolmerPatricia P. WadowskiDaniel Marcelo CisternaPetar OzretićDaniele CuntreraPhoebe BarnettDimitrios KaragiannakisPietro C. DattoloDivya KandariPilar GiraldoDominik DłuskiPing-Chih HsuDominika KalankovaPiotr YablonskiiDong-Hyun KimPooneh SalariDragoș Ioan TohăneanPrahlad HoDragos Paul MihaiPritam SadhukhanDulce Maria MinayaPurva V. SharmaEduard Maury-SintjagoRafael Moreno-Gómez-ToledanoEduardo Gutiérrez-AbejónRaffaello PellegrinoEleanor WilsonRam Vinay PandeyElena KozhevnikovaRavindra DeshpandeElena-Mihaela CordeanuRazvan BardanEmma MontellaRenato PietrolettiEmmanuel AndresReza MortazaviErik KaestnerRio Ryohichi SugimuraEsma ÖzkanRocio Alejandra Chavez-SantoscoyEugenio PaciRocío Llamas-RamosEvasio PasiniRodolfo RedaFarzad Taghizadeh-HesaryRosa DivellaFederica Di SpiritoRossana IzzettiFederico RoggioRyan Ruiyang LingFilippo GaviSaeed KabrahFilippo LucianiSarhang HamaFlavia IaculliSarika GuptaFrancesco BiancoSarvath AliFrancesco MassoniSayed Samed TalibiFrancisca García-Moreno NisaSeason S. WongGašper KlančarSebastian SchnaubeltGemma Caterina Maria RossiSebastián Valverde-MartínezGeorge ImatakaSepideh HosseiniporghamGiancarlo RussoSeyed Arash AlawiGiulio Francesco RomitiShadma W. AbdulwahabGiuseppe AuteriShankargouda PatilGöran KurlbergShilpa BishtGulzhanat AimagambetovaShilpa GopinathGyehyun JungShuhua ZhengHariyono WinartoSimone RanaldiHasriadi HasriadiSokol TrunguHiroya KuroyanagiSousana PapadopoulouHussein AlmasriSreenath NairHyo-Jung JeongSriyani PadmalathaIbtihal FadhilStanisław HorakIgor DumicStoyan KostovIoannis VentoulisSylvère StörmannIrina NovikovaTaiwo AremuIvana JochmanováTakaaki SenbonmatsuJahanpour AlipourTetsuo NishikawaJan Von Der ThüsenThomas EmmanuelJavier Carbajo-LozoyaTony KuoJelena RoganovicUlf NestlerJeong HeoUrmi KhannaJoanna Popiołek-KaliszUrsula CatenaJonathan BayuoValene Garr BarryJosé D. MéndezValeria ViscoJoshua Porat-DahlerbruchVeljko DubljevićJuan-Manuel Praena-FernándezViktor DomisloviĆJulio Plaza DíazVipul D. YagnikKaila L. StipancicVittorio ChecchiKamil KoszelaWaldemar PlacekKarin MagnussenWenchun ChenKazuhiko KurozumiWi-Young SoKelly McGormWouter KokKen HillmanXuejing YuKeyur VoraXulong ZhangKoichi NaitoYasuhiro KuroiKovy Arteaga-LiviasYasuhiro MochidaKrupa NavalkarYasushi ShibataKrzysztof GomułkaYawei LiKuan-Hui ChenYen Po WangKyuseok KimYen-Chien LeeLadislav StankeYiming LiangLaetitia ChauviereYoshihiro FukumotoLars HeggelundYudai IshiyamaLeon SeifertYulia O. BobrovaLinda V. GraudinsYvonne SingerLingpeng ShanZbigniew AdamiakLuca CimaZhe JinLucia StančiakováZoe Valero-Ramón


